# Hipertensão na Adolescência, uma Relação Direta com Obesidade e Resistência à Insulina

**DOI:** 10.36660/abc.20220188

**Published:** 2022-04-07

**Authors:** Mario Fritsch Neves

**Affiliations:** 1 Universidade do Estado do Rio de Janeiro Rio de Janeiro Brasil Universidade do Estado do Rio de Janeiro - Clínica Médica, Rio de Janeiro, RJ – Brasil

**Keywords:** Adolescente, Hipertensão, Obesidade, Fatores de Risco, Resistência à Insulina, Sedentarismo, Epidemiologia

A hipertensão arterial é o principal fator de risco modificável para o desenvolvimento de doenças cardiovasculares e sua ocorrência em idades mais precoces favorece o envelhecimento vascular acelerado nos anos seguintes.^1^ O aumento da pressão arterial na adolescência geralmente não ocorre de forma isolada e está associada a outros fatores de risco como ingestão excessiva de sal, atividade física reduzida e, principalmente, sobrepeso/obesidade.^2,3^ Considerando que um elevado percentual de gordura na infância e na adolescência tem efeitos adversos precoces sobre a pressão arterial, medidas adequadas da gordura corporal podem determinar marcadores mais precisos de maior adiposidade e preditores da incidência de hipertensão nos indivíduos mais jovens.

Grandes avanços da tecnologia estão presentes na vida diária dos adolescentes e geralmente favorecem a inatividade física e o ganho de peso que apresenta relação direta com os níveis de pressão arterial. Além do sedentarismo estar fortemente associado à hipertensão na adolescência, o exercício físico exerce um papel protetor, reduzindo a pressão arterial por vários mecanismos. Estudo transversal realizado com crianças e adolescentes de 11 a 17 anos demonstrou a associação do sexo masculino e obesidade central com hipertensão nesses escolares. Por outro lado, o mesmo estudo apontou a prática de atividades físicas moderadas e vigorosas como forma eficaz de prevenir a elevação da pressão arterial diastólica nos jovens desta idade.^4^

O desequilíbrio autonômico parece ser um dos mecanismos iniciais para a elevação da pressão arterial em adolescentes. Neste grupo de indivíduos jovens, o desequilíbrio autonômico é representado principalmente pela hiperatividade simpática, que por sua vez, também está associada com obesidade, alterações do padrão do sono e, consequentemente, maior risco de eventos cardiovasculares. Um estudo recente demonstrou que adolescentes, mesmo na faixa de pré-hipertensão, já apresentam disfunção autonômica avaliada pela variabilidade da frequência cardíaca.^5^

No estudo realizado por Lima et al.,^6^ publicado nesta edição dos Arquivos Brasileiros de Cardiologia, os autores procuraram determinar a prevalência de hipertensão e a sua associação com o perfil lipídico, glicídico e de adiposidade. A originalidade deste projeto é que foi realizado numa população de 1200 adolescentes, de 12 a 17 anos, do Distrito Federal que foram participantes do Estudo de Riscos Cardiovasculares em Adolescentes (ERICA).^7^ A prevalência de 8% de hipertensão encontrada entre os adolescentes do DF foi semelhante a outras regiões geográficas do país avaliadas no mesmo estudo ERICA, exceto a região sul com prevalência de 12,5%, bem acima do estudo atual e das demais regiões. Os adolescentes hipertensos apresentaram parâmetros mais elevados de adiposidade e maior ocorrência de hiperinsulinemia, mas a alteração mais encontrada foram os baixos níveis de HDL-colesterol. A maioria das variáveis se correlacionou com os níveis de pressão arterial sistólica e diastólica e, mesmo após ajustes, o índice de massa corporal (IMC) e o modelo de avaliação da homeostase da resistência à insulina (HOMA-IR) foram os parâmetros com maior força de associação.

Avaliação de resistência à insulina em adolescentes é um grande desafio. Nesta faixa etária, os níveis de insulina costumam ser mais elevados e associados a outras alterações hormonais relacionadas com modificações corporais. Entretanto, essa não parece ser a justificativa para hiperinsulinemia relatada no presente estudo, pois os autores indicam que os adolescentes, na sua grande maioria, já estavam no final da puberdade. Além disso, os adolescentes não hipertensos apresentaram níveis de insulina substancialmente menores do que a aqueles com hipertensão, sugerindo uma relação mais direta. Neste caso, a resistência à insulina pode ser a confirmação bioquímica da síndrome metabólica, condição que vem aumentando de frequência na infância e na adolescência, determinando maior risco de aparecimento de doenças crônicas na vida adulta.^8^

A maioria dos estudos clínicos com hipertensão é com participantes adultos e/ou idosos. No Brasil e no mundo, há poucas publicações referentes à hipertensão em adolescentes. Isso reforça a importância desse estudo, pois precisamos de dados nacionais que servirão de base para nossas futuras diretrizes nessa área. Certamente o Distrito Federal não representa a realidade de todo nosso país, o que limita a validade externa e com isso, não podemos extrapolar os resultados atuais. Por outro lado, os achados indicam importantes informações que se juntam aos outros estudos no Brasil e, dessa forma, podemos construir um painel nacional mais confiável.
