# Supplementary material for: Hipertensão na Adolescência, uma Relação Direta com Obesidade e Resistência à Insulina
Source: Arq Bras Cardiol. 2022 Apr 7;118(4):727–9. [Article in Portuguese] doi: 10.36660/abc.20220188 (PMC9007015; doi:10.36660/abc.20220188)
Supplement: Translation

# Translation: Hypertension in Adolescence, a Direct Relationship to Obesity and Insulin Resistance

**Authors:** Mario Fritsch Neves

**Affiliations:** 1 Brazil Universidade do Estado do Rio de Janeiro - Clínica Médica, Rio de Janeiro, RJ – Brazil

**Keywords:** Adolescent, Hypertension, Obesity, Risk Factors, Insulin Resistance, Sedentarism, Epidemiology

Hypertension is the main modifiable risk factor for developing cardiovascular diseases, and its occurrence at an earlier age favors accelerated vascular aging in the following years.^1^ The increase in blood pressure in adolescence does not usually occur in isolation and is associated with other risk factors such as excessive salt intake, reduced physical activity, and, especially, overweight/obesity.^2,3^ Considering that a high-fat percentage in childhood and adolescence has early adverse effects on blood pressure, adequate body fat measurements can determine more accurate markers of higher adiposity and predictors of the incidence of hypertension in younger individuals.

Major advances in technology are present in adolescents’ daily lives and generally favor physical inactivity and weight gain, which are directly related to blood pressure levels. In addition to a sedentary lifestyle strongly associated with hypertension in adolescence, physical exercise plays a protective role, reducing blood pressure by several mechanisms. A cross-sectional study with children and adolescents aged 11 to 17 years showed the association of male gender and central obesity with hypertension in these students. On the other hand, the same study pointed to moderate and vigorous physical activities as an effective way to prevent the increase in diastolic blood pressure in young people of this age.^4^

Autonomic imbalance seems to be one of the initial mechanisms for blood pressure elevation in adolescents. In this group of young individuals, the autonomic imbalance is mainly represented by sympathetic hyperactivity, which is also associated with obesity, changes in sleep patterns and, consequently, an increased risk of cardiovascular events. A recent study has shown that adolescents already have autonomic dysfunction assessed by heart rate variability, even in the pre-hypertension range.^5^

In the study carried out by Lima et al.,^6^ published in this issue of *Arquivos Brasileiros de Cardiologia*, the authors intended to determine the prevalence of hypertension and its association with lipid, glucose and adiposity profiles. The originality of this project is that it was carried out in a population of 1200 adolescents, aged 12 to 17 years, from the Federal District who participated in the Study of Cardiovascular Risks in Adolescents (ERICA).^7^ The 8% prevalence of hypertension found among adolescents in the Federal District was similar to the other geographic regions of the country evaluated in the same ERICA study, except for the southern region with a prevalence of 12.5%, well above the current study and the other regions. Hypertensive adolescents had higher adiposity parameters and a higher occurrence of hyperinsulinemia, but the most common alteration was low levels of HDL-cholesterol. Most variables correlated with systolic and diastolic blood pressure levels and, even after adjustments, body mass index (BMI) and the homeostatic model assessment for insulin resistance (HOMA-IR) were the parameters with the highest association strength.

Assessment of insulin resistance in adolescents is a major challenge. In this age group, insulin levels tend to be higher and associated with other hormonal changes related to body modifications. However, this does not seem to be the reason for the hyperinsulinemia reported in the present study, as the authors indicate that most adolescents were already at the end of puberty. In addition, non-hypertensive adolescents had substantially lower insulin levels than those with hypertension, suggesting a more direct relationship. In this case, insulin resistance may be the biochemical confirmation of the metabolic syndrome, a condition increasing in frequency in childhood and adolescence, leading to a greater risk of developing chronic diseases in adulthood.^8^

Most clinical trials with hypertension involve the adult and/or elderly participants. In Brazil and worldwide, there are few publications regarding hypertension in adolescents. This reinforces the importance of this study, as we need national data that will form the basis for our future guidelines in this area. Certainly, the Federal District does not represent the reality of our entire country, which limits the external validity and, therefore, we cannot extrapolate the current results. On the other hand, the findings indicate important information that adds to other studies in Brazil, and, in this way, we can build a more reliable national panel.
